# Lithium in drinking water and crime rates in Japan: cross-sectional study

**DOI:** 10.1192/bjo.2020.63

**Published:** 2020-10-15

**Authors:** Kentaro Kohno, Nobuyoshi Ishii, Hirofumi Hirakawa, Takeshi Terao

**Affiliations:** Department of Neuropsychiatry, Faculty of Medicine, Oita University, Yufu City, Japan; Department of Neuropsychiatry, Faculty of Medicine, Oita University, Yufu City, Japan; Department of Neuropsychiatry, Faculty of Medicine, Oita University, Yufu City, Japan; Department of Neuropsychiatry, Faculty of Medicine, Oita University, Yufu City, Japan

**Keywords:** Lithium, trace element, drinking water, crime, epidemiological study

## Abstract

**Background:**

In pharmacological doses, lithium successfully treats bipolar disorder and it can reduce violent crimes committed by individuals with this disorder.

**Aims:**

To investigate whether naturally occurring lithium in drinking water lowers rates of violent crime in the general population.

**Method:**

We examined lithium levels in the drinking water of the 274 municipalities of Kyushu Island in Japan and compared these with the crime rates in each municipality.

**Results:**

We found that lithium levels were significantly and inversely associated with crime rates in 2009.

**Conclusions:**

Our findings suggest that even very low levels of lithium in drinking water may play a role in reducing crime rates in the general population.

Lithium is a soft, silvery white alkali metal that occurs naturally in foods (such as grains and vegetables). Significant amounts may also occur in drinking water. Research shows that it is probably essential to human health and it has been suggested that a 70 kg adult should aim for a dietary intake of 1000 μg/day.^1^

Generally accepted as a first-line treatment for bipolar disorder, lithium is also thought to be one of the best augmenting therapies for treatment-resistant depression.^2^ Meta-analysis of randomised controlled trials^2^ found lithium to be significantly more effective than placebo in reducing the number of suicides and all-cause mortality in the long-term treatment of mood disorders. Meta-analysis^4^ also suggests that lithium therapy can improve cognitive performance in mild cognitive impairment and Alzheimer's disease. Finally, a meta-analysis^5^ showed that lithium was significantly better than placebo in treating aggression. Thus, lithium is a very useful psychotropic medication.

A number of studies, mostly from the past decade, have investigated the effects of very small amounts of lithium in treating suicidality^6–10^ and dementia^11,12^ and in modifying temperament^13,14^ and criminal behaviour.^6,15–17^ They suggest that trace lithium might be an effective prophylaxis for various psychiatric conditions, but randomised controlled trials are needed to enable definite conclusions to be drawn. The use of trace lithium to treat conditions such as dementia or to modify temperament or criminal behavioural is supported by very limited evidence and its effects are yet to be confirmed.^2^

This paper is part of a study of various potential effects of lithium in drinking water on a large population in Japan.^7,8^ Here we report on the association between lithium levels and crime rates in each of the municipalities of Kyushu Island, Japan.

## Method

### Study population

Kyushu Island is the southernmost of Japan's four large islands. In 2009, the total population of Kyushu Island was 14 684 991. Kyushu Island has 118 cities, 119 towns and 37 villages, totalling 274 municipalities. Of the 274 municipalities, Fukuoka City had the largest population (1 384 820 residents) and Mishima village had the smallest (368 residents). This large difference in population was reflected across all 274 municipalities.^8^ These data were acquired from the Ministry of Internal Affairs and Communications, Tokyo, Japan. We assert that all procedures contributing to this work comply with the ethical standards of the relevant national and institutional committees on human experimentation and with the Helsinki Declaration of 1975, as revised in 2008.

### Measurement of lithium levels in drinking water

Between 2010 and 2013, 434 drinking water samples were taken in the 274 municipalities (mainly from the main railway station or the municipal offices) and their lithium levels were measured by a third party using mass spectroscopy. This method can measure lithium levels as low as 0.1 parts per billion (0.1 μg/L). Where samples were taken at multiple points in the same municipality, the mean value was calculated. Although only single samples were taken at each location, resampling in the same places 1 year later confirmed that there was very little fluctuation in levels (correlation coefficient: 0.998).^8^

### Crime rates

The criminal offences evaluated included homicide, rape, robbery, arson, violence, fraud and gambling, but excluded negligence resulting in traffic accidents. Crime rates were calculated by dividing the number of recognised criminal offences by the total populations per municipality in 2009, because crime data have not been published since then.

### Adjustment factors

We first generated a crude model of the association between lithium levels in drinking water and crime rates, without adjusting for confounding factors (crude model). We then studied the associations in more detail by adjusting for the proportion of 1-person households,^18^ the overall unemployment rate^19^ and the proportion of elderly people^20^ (adjusted model). These data were available for 2010 for all 274 municipalities (from the Statistics Bureau, Ministry of Internal Affairs and Communications).

### Statistical analysis

Treating all data equally would give less precisely measured data points more influence than they merit and highly precise data points too little influence. We therefore performed a weighted least squares regression analysis in an attempt to give each data point its proper influence over the parameter estimates. To allow for the great differences in population size across the 274 municipalities, we also used weighted least squares regression analysis adjusted for the size of each population.^8^ We used multiple regression analyses to calculate crime rates in 2009 from lithium levels and the adjustment factors in 2010.

## Results

### Lithium levels

**Fig. 1:**
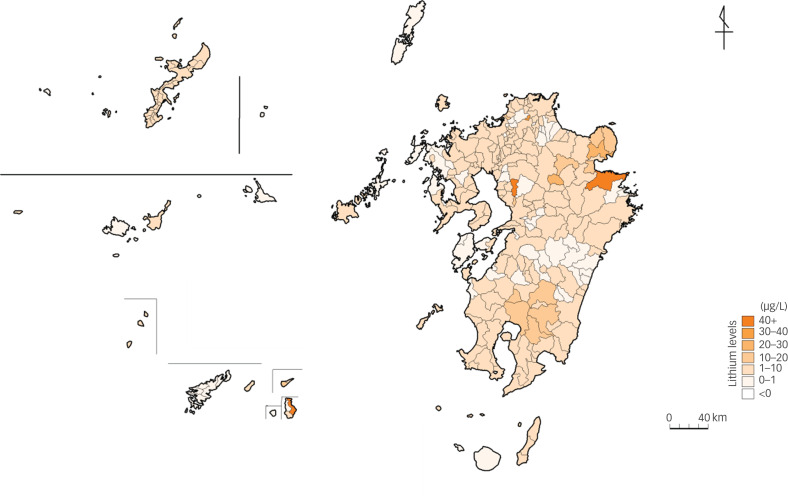
Lithium levels in drinking water across the 274 municipalities of Kyushu Island, Japan (2010–2013).

Figure 1 shows the lithium levels in the drinking water across Kyushu Island's 274 municipalities from 2010 to 2013. The mean level was 4.2 μg/L (s.d. = 9.3; range 0–130).

### Crime data

**Fig. 2:**
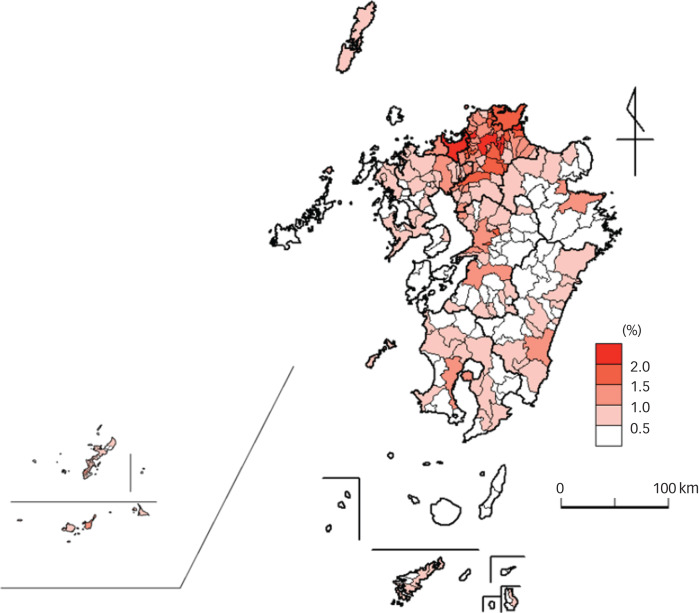
Crime rates across the 274 municipalities of Kyushu Island, Japan (2009).

The National Police Agency recognised 166 056 criminal offences across Kyushu Island in 2009 (from the Statistics Bureau, Ministry of Internal Affairs and Communications).^21^ Figure 2 shows the crime rates across Kyushu Island's 274 municipalities. The mean crime rate was 1.13%.

### Adjustment factors

In 2010, the mean proportion of 1-person households in 2010 was 27.2% (s.d. = 7.6; range 14.4–61.6). The mean overall unemployment rate was 7.5% (s.d. = 2.9; range 0.8–20.3). The mean proportion of elderly people was 27.7% (s.d. = 6.4; range 14.0–43.4).^8^

### Association between crime rates and lithium levels

As shown in Table 1, we found no association between lithium levels in drinking water and crime rates in the crude model. However, lithium levels were significantly and inversely associated with crime rates after adjusting for the proportion of elderly people, the proportion of 1-person households and the overall unemployment rate (adjusted model). There was no multicollinearity in the multiple regression analyses.

**Table 1:** Crime rates and lithium levels in drinking water (274 municipalities of Kyushu Island, Japan)

Model	Lithium levels		
	β	*P*	Adjusted *R*^2^
Crude model	−0.100	0.100	0.006
Adjusted model	−0.106	0.016	0.482
Adjusted for:			
Proportion of 1-person households	0.442	0.000	
Overall unemployment rate	0.076	0.092	
Proportion of elderly people	−0.387	0.000	

## Discussion

It has been suggested that high impulsivity is a risk factor for criminal involvement,^22^ and both animal and human studies have examined the effects of lithium on impulsivity. Ohmura *et al* ^23^ reported that lithium (but not valproate or carbamazepine) suppressed impulsive actions in rats, and Halcomb *et al* ^24^ reported that lithium (but not valproate) reduced impulsive choice in the delay-discounting task in mice. Moreover, lithium decreases levels of impulsivity as measured by various outcome measures not only in individuals with bipolar disorder but also in those with other impulse control disorders.^5,25^

As far as we know, the first evidence that trace amounts of lithium in tap water might affect mental health appeared in 1972, when Dawson *et al* reported that psychiatric hospital admissions and homicide rates were lower in Texas counties with higher lithium levels in the water supply.^15^ In 1990, Schrauzer & Shrestha^6^ published a study examining the association between lithium in drinking water and crime, suicide and drug-related arrests, again based in Texas. Water samples were taken in 27 Texas counties between 1978 and 1987. The mean annual incidence rates of homicide, suicide, rape, robbery, burglary, theft and total crime over the sampling period were significantly higher in counties with low lithium levels (mean 5 μg/L; s.d. = 4; range 0–12) than in those with high levels (mean 123 μg/L; s.d. = 25; range 70–160); all differences except those for assault were statistically significant (*P* < 0.01). The authors concluded that their results suggest that lithium at levels that may be encountered in public water supplies has moderating effects on suicidal and violent criminal behaviour. They subsequently reported that lithium levels in scalp hair are low in incarcerated violent criminals.^16^ In Greece, Giotakos *et al*^17^ reported that the mean number of homicides tended to be lower in prefectures with high levels of lithium in the water supply (*R*^2^ = 0.054, β = −0.38, *P* = 0.004). Ando *et al* ^26^ investigated the association between lithium levels in tap water and mental health problems in a general population of adolescents using a large individual-level data-set in Japan between 2008 and 2009. They found an inverse association between lithium levels (mean 0.48 μg/L; s.d. = 0.52; range 0.01–2.10) and both symptoms of depression (*P* = 0.02) and interpersonal violence (*P* = 0.02).

Unlike the Texas study,^5^ in our study we used lithium levels as a continuous value, to exclude the possibility of spurious findings resulting from the arbitrary division of the levels. We found that lithium levels were significantly and inversely associated with crime rates across the 274 municipalities in Kyushu Island in the adjusted model.

### Limitations and further research

This study has several limitations. The first is methodological problems. Although we used multiple regression analyses, it is necessary to consider whether an alternative statistical method (such as nonlinear model) would be more appropriate in future analyses. Second, we did not gather data on lithium intake from food, which generally exceeds that from drinking water.^27^ Goldstein & Mascitelli^27^ suggested that, to ensure adequate daily dietary intake of lithium for the purpose of decreasing violence and aggression, nations should consider fortifying cereal grain products with lithium and adding lithium to vitamin preparations for adults. Third, bipolar disorder is deeply linked to impulsive crime. We should investigate whether the effects on crime of lithium in drinking water might be extrapolated from results on bipolar disorder. Regrettably, we could not obtain prevalence data on bipolar disorder in the different municipalities in Japan. However, in a nationwide Danish study, Kessing *et al* ^28^ reported that the median of the average lithium exposure did not differ between individuals with mania/bipolar disorder (12.7 μg/L; interquartile range IQR = 7.9–15.5 μg/L) and age- and gender-matched controls (12.5 μg/L; IQR = 7.6–15.7 μg/L; *P* = 0.2) and that the incidence rate ratio of mania/bipolar disorder did not decrease with higher long-term lithium exposure overall or within age groups (0–40, 41–60 and 61–100 years of age). Therefore, lithium intake from drinking water may be not associated with the incidence rate of bipolar disorder. Finally, we did not collect data on the study population's consumption of mineral water. In a previous study, we found that lithium levels in foreign mineral waters were significantly higher than those in Japanese mineral waters and tap water, although there was no significant difference between Japanese mineral waters and tap water.^29^ Future studies might consider what types of mineral water participants drink, to allow adjustment of the analytical data on the potential effect of lithium on crime. Further studies are also required to confirm the findings presented in this paper and to investigate gender differences in the suggested association between lithium in drinking water and crime rates.

## Data Availability

Authors have access to the original study data.
